# Reconfigurable nanoscale spin-wave directional coupler using spin-orbit torque

**DOI:** 10.1038/s41598-019-43597-6

**Published:** 2019-05-08

**Authors:** Zhiwei Ren, Shuang Liu, Lichuan Jin, Tianlong Wen, Yulong Liao, Xiaoli Tang, Huaiwu Zhang, Zhiyong Zhong

**Affiliations:** State Key Laboratory of Electronic Thin Films and Integrated Devices, University of Electronic Science and Technology of China, Chengdu, 611731 China

**Keywords:** Electronics, photonics and device physics, Spintronics

## Abstract

We present a reconfigurable nanoscale spin-wave directional coupler based on spin-orbit torque (SOT). By micromagnetic simulations, it is demonstrated that the functionality and operating frequency of proposed device can be dynamically switched by inverting the whole or part of the relative magnetic configuration of the dipolar-coupled waveguides using SOT. Utilizing the effect of sudden change in coupling length, the functionality of power divider can be realized. The proposed reconfigurable spin-wave directional coupler opens a way for two-dimensional planar magnonic integrated circuits.

## Introduction

Information encoded in spin waves (SWs) can be transmitted and processed without the flow of electrons. This low power consumption feature with high operational speed makes SWs become one of the most promising alternatives to CMOS technology^1–6^. A typical SW-based signal processing system consists of SW excitation, detection and functional components^1,2,4–10^. Among numerous novel SW functional devices, SW couplers are of special importance owing to their promising application as efficient and controllable connection between functional components in magnonic circuits^11–15^. At the same time, they can also be used to realize power divider, multiplexer, etc.^12,14^. In addition, SW couplers which comprise a system of laterally coupled magnonic crystals present many interesting nonlinear SW phenomena^16–19^.

In recent years, reconfigurable SW devices arouse tremendous interest for its functionality can be tuned dynamically for improving the efficiency and flexibility of magnonic integrated circuits^20–24^. The variation of electric or/and bias magnetic field offers the possibility to realize reconfigurable SW couplers^12,14,15^. However, for electric-field adjustment method, the need of hundreds-microns-thick of ferroelectric ceramic layer is not suitable for future planar magnonic circuits. For the method based on bias magnetic field, the requirement of continuous energy supply during operation is inefficient. Qi Wang *et al*. proposed a way to switch the functionality of SW couplers by inverting the relative magnetic configuration of the coupled waveguides^14^. Once the magnetic configuration is inverted, the functionality of the coupler can be switched and the energy supply can be shut off. But the method by which they realize the magnetic configuration inversion is applying magnetic field pulse. The introduction of magnetic field generator would significantly increase the volume of circuits, and further the non-localized magnetic field can easily affect other around devices.

To solve this problem, the current-induced spin-orbit torque (SOT) can be utilized^25^. In the past few years, the use of SOT as the tool for switching magnetization has been widely explored^26–28^. This effect origins from the strong spin-orbit coupling at the bulk of heavy metals and/or the interface of heavy metal/ferromagnet. Unlike traditional spin transfer torque (STT), in SOT case the electrons do not need to pass through the magnetic layer. Thus, SOT is highly efficient and can be used in the occasion even where the magnetic material is insulator, for instance, the popular material for SW devices — YIG (Y_3_Fe_5_O_12_).

Here, we propose a reconfigurable nanoscale SW directional coupler based on current-induced SOT. The functionality and operating frequency of proposed coupler can be dynamically switched by inverting the magnetic configuration of one of the coupled waveguides using SOT.

## Results and Discussion

**Figure 1:**
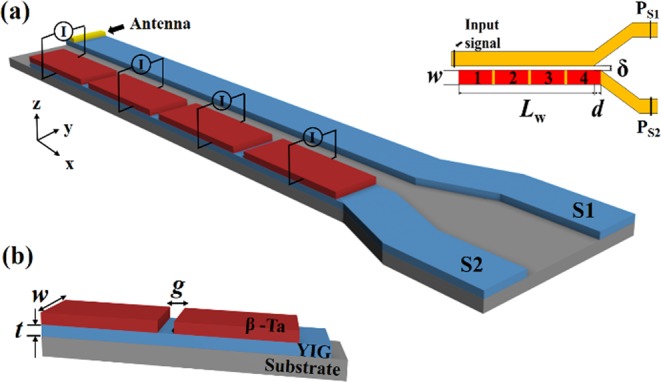
(**a**) Schematic of the reconfigurable nanoscale spin-wave directional coupler based on SOT. The working length of the coupled waveguides is *L*_w_ = 8 μm, translational shift *d* = 100 nm, the widths of the waveguides are *w* = 100 nm, gap δ = 30 nm and the regions covered by heavy metal are numbered as shown in the top view. The SWs are excited in S1. (**b**) Close-up view of S2. A YIG waveguide of *t* = 30 nm thickness is placed on the substrate. The coupled part of S2 is covered by identical β-Ta strips which are separated by *g* = 20 nm from each other. For switching magnetic configuration, current is applied on β-Ta strips in y-direction in order to generate pure spin current that polarized towards x-direction.

The schematic of the proposed device is shown in Fig. 1(a,b). Two parallel YIG waveguides S1 and S2 with width *w* = 100 nm and thickness *t* = 30 nm are placed laterally parallel with a gap δ = 30 nm. The working length is *L*_w_ = 8 μm. A translational shift *d* = 100 nm is introduced between two waveguides in order to minimize the secondary SW sources effect of the corners^14^. Four identical β-Ta strips which are separated by a gap with width of *g* = 20 nm from each other are placed on the S2 waveguide. They are numbered as shown in the top view of the model (see Fig. 1(a)). The initial magnetization direction is along the +x direction. Electrical current is applied on β-Ta strips in the y-direction to flip the magnetic configuration in S2. To excite a propagating SW in S1, a sinusoidal magnetic field *b*_*y*_ = *b*_0_ sin (2*πft*) with the amplitude *b*_0_ = 1 mT produced by 20 nm width microstripe antenna is applied at the edge of S1. The SW power is output via ports shown in the top view of Fig. 1(a).

**Figure 2:**
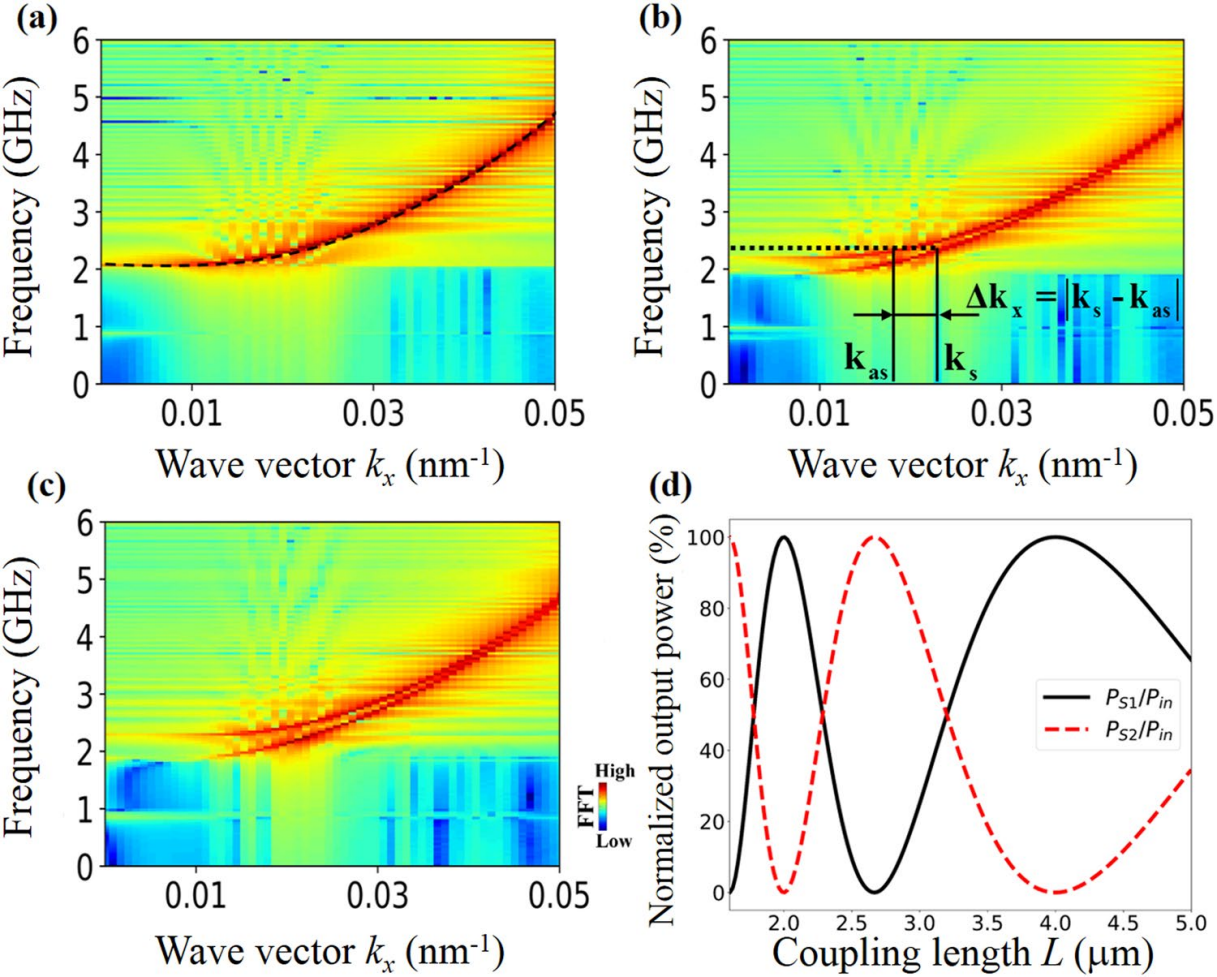
(**a**–**c**) Dispersion characteristics of the lowest width SW modes in different conditions. (**a**) Dispersion characteristics in a single isolated waveguide. A color map represents the simulation result, whereas the dashed line represents the theoretical calculation result according to the ref.^10^. (**b**,**c**) Dispersion curves in a pair of coupled waveguides in parallel and antiparallel magnetic states, respectively. (**d**) The dependence of normalized output powers of two coupled waveguides on coupling length *L*. The Gilbert damping is ignored.

First, we elucidate the working principle of typical SW directional couplers. Owing to the dipolar coupling between the two waveguides, the lowest width SW mode in one single waveguide is split into two collective modes — symmetric and antisymmetric mode (see Fig. 2(a,b))^12–15,29^. Therefore, two collective SW modes are excited simultaneously with different wave numbers *k*_s_ and *k*_as_ in a coupler (*k*_s_ and *k*_as_ for symmetric and antisymmetric mode, respectively). The interference between these two modes results in the periodic energy transfer between the two waveguides. The spatial period of energy transfer along the propagation direction is defined as coupling length *L*^29^.

1 $$L={\rm{\pi }}/|{k}_{s}-{k}_{as}|$$

The output powers of the two waveguides can be expressed as following^29^: ${P}_{{\rm{S}}1}={P}_{in}{\cos }^{2}(\pi {L}_{w}/(2L))$ and ${P}_{{\rm{S}}2}={P}_{in}{\sin }^{2}(\pi {L}_{w}/(2L))$ Where *P*_in_ is the input power. The normalized output powers of the two waveguides can be expressed as:

2 $$\begin{array}{rcl}{P}_{{\rm{S}}1}/{P}_{in} & = & {\cos }^{2}(\pi {L}_{w}/(2L))\\ {P}_{{\rm{S}}2}/{P}_{in} & = & {\sin }^{2}(\pi {L}_{w}/(2L))\end{array}$$

These dependencies are shown in Fig. 2(d) without considering damping. With Eq. (1), one can know that the output powers of waveguides are determined by the wave-number difference ${\rm{\Delta }}k=|{k}_{s}-{k}_{as}|$, i.e. the splitting degree in the dispersion curve. However, for nanoscale waveguides, there are two relative magnetic configurations — parallel and antiparallel static magnetic states — due to the strong shape anisotropy along the long-axis in the absence of bias field. The dispersion curve of the lowest width mode in the case of antiparallel magnetization configuration is depicted in Fig. 2(c). By comparing with Fig. 2(b,c), one can find that the splitting degree in dispersion curve in antiparallel case is stronger than that in parallel case. Thus, the coupling length *L* is always shorter in antiparallel state than that in parallel state for a specific SW. Since *L* can be modified by inverting magnetization configuration, the functionality can be switched by the same way. For example, according to Eq. (2), if the *L* satisfies the condition *L*_*w*_ = 2*nL* in parallel state for a specific SW, where n is an integer, the entire energy will be transferred back to S1 at the end. The coupler acts as a transmission line. Whereas in antiparallel state, the *L* for the same SW is shorter, if the *L* satisfies the condition *L*_*w*_ = (2*n* + 1)*L*, the energy will be totally transferred to S2. Now the coupler can act as a connector of magnonic conduits.

**Figure 3:**
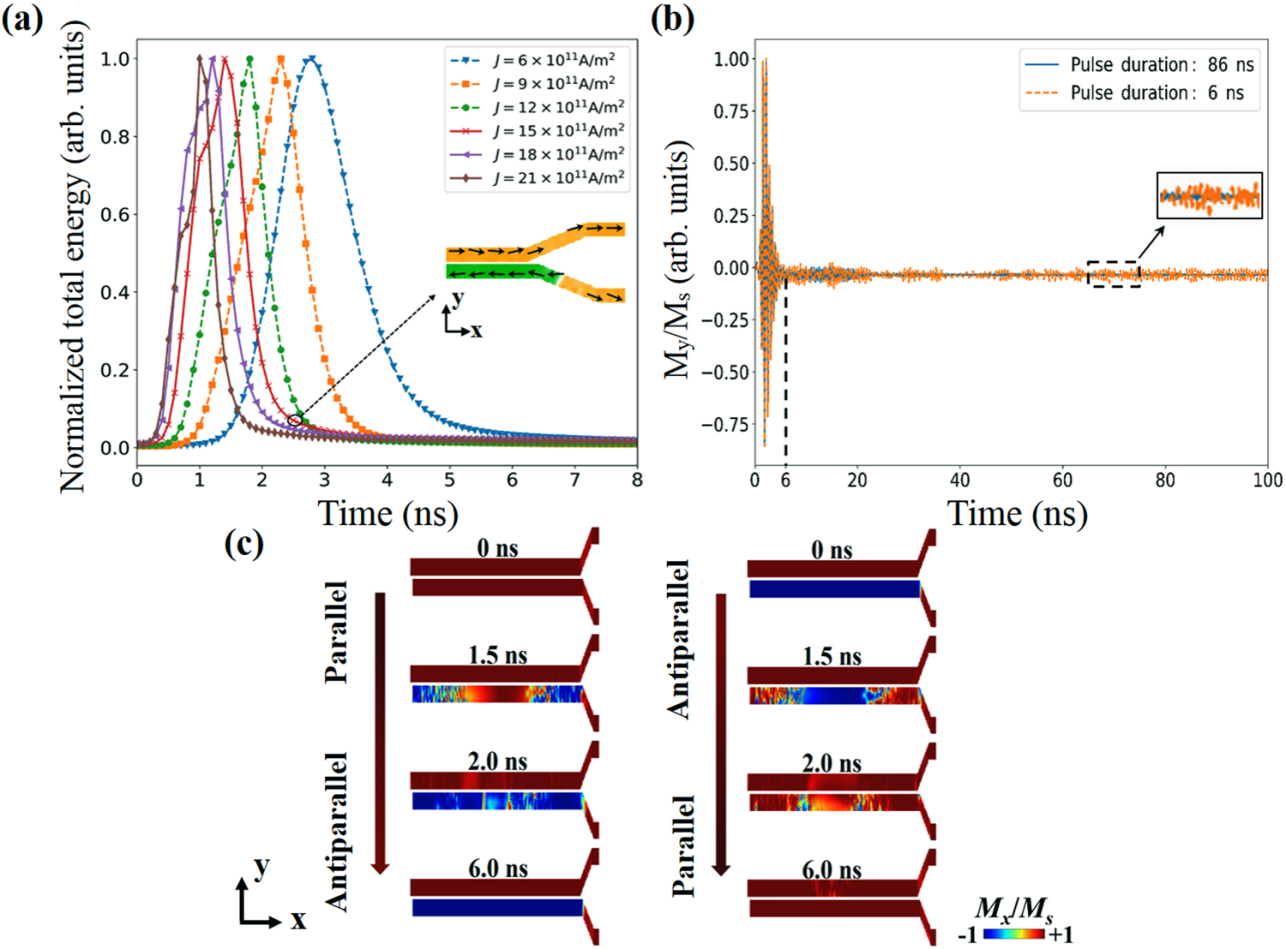
(**a**) Dependence of normalized total free energy in the coupler on electric current applied time. Data are normalized by dividing the maximum of each case. Current is applied on all four β-Ta strips in +y direction. Inset: the snapshot of coupler magnetic configuration. The selected moment is 2.7 ns, and current density *J* = 15 × 10^11^ A/m^2^. The arrows show the direction of magnetization. (**b**) The time evolution of the average *M*_y_/*M*_s_ with different current pulse duration. (**c**) Snapshots of the magnetic configuration inversion processes of directional coupler. Current is applied on all four β-Ta strips in ±y directions, respectively. The applied current density *J* = 12 × 10^11^ A/m^2^.

Next, in order to elaborate the mechanism of SOT-induced magnetic configuration inversion and determine the optimal current density, we describe the inversion process from an energy perspective. The method used here is based on current-induced SOT^25^. The charge current is applied on β-Ta strips in the y-direction, then as the result of the strong spin-orbit coupling in β-Ta, the pure spin current that polarized towards the x-direction is generated and flows into the YIG film (directions in Fig. 1(a)). Finally, magnetic moments can be flipped by the torque exerted by this spin current^26–28^. Figure 3(a) shows the normalized total free energy in the coupler dependent on current applied time for different current densities when current is applied on all four metal strips in the +y direction. The threshold inverting current density is *J* = 6 × 10^11^ A/m^2^. In general, the inversion can complete in 8 ns and the speed is proportional to the magnitude of current density. However, as shown in the inset of Fig. 3(a), when the value of current density is too high (this critical current density is 15 × 10^11^ A/m^2^ in our case), the inversion region will expand into the area that is not covered by metal. It is owing to the over-strong spin torque resulted by high current density. This situation is harmful to the reversion operation. Therefore, the current density is determined to *J* = 12 × 10^11^ A/m^2^. Since the inappropriate design of the geometry of the coupler may raise a further increase of the current density, it is worthy to note the tradeoff between the current density and the size of the coupler. In general, due to the shape anisotropy, the threshold current density is proportional to the ratio of *L*_w_ to *w*. But for the bias-free propagation of spin wave, this ratio cannot be too small. Additionally, since SOT is an interfacial phenomenon, the increase of the thickness of YIG film may increase the threshold current density. However, the decrease of the thickness will lead to the reduction of the coupling efficiency and consequently increase *L*_w_^14^. Thus, when design a proposed coupler one should balance all of geometric factors mentioned above. To reduce the current density, using material with larger spin Hall angle, such as β-W is also an approach^30^. But it may result in an increase of the damping in YIG waveguide, and consequently intensify the dissipation of spin wave power.

To demonstrate the effective inversion and reversion of magnetic configuration, we plot the snapshots of the processes. Figure 3(c) show the temporal evolution of the magnetic configuration when current is applied on all four metal strips with *J* = 12 × 10^11^ A/m^2^ in the ±y directions. The inversion of S2 finishes in 6 ns, but this evolution results in the excitation of parasitic SWs in S1 (see the long tail in *M*_y_ characteristics in Fig. 3(b)). This lead to the device is ready for operation after a time period exceeding one SW life cycle. To speed up the relaxation, the pulse duration is extended to 86 ns (see Fig. 3(b)). After shutting off the current, it takes additional 70 ns to obtain ground-state configuration, i.e. the total time cost is 156 ns. It is much shorter than the case using magnetic field (at least 282 ns)^14^. There are two principles can explain the acceleration of the relaxation. On one hand, continuous application of the torque in x-direction can fix the moments in S2. Consequently, the excitation of parasitic SWs in S2 can be avoided. On the other hand, fixed moments in S2 can “hitch” the moments in S1 due to the dipolar coupling between these two waveguides. Thereby it can speed up the attenuation of parasitic SWs in S1. One may notice that the inversion starts from both ends of the coupled part of S2, then expands to the center rather than happens simultaneously. It is because the magnetic moments at the ends of a waveguide are not strictly parallel to the long-axis in order to meet the principle of energy minimum. Thus, there are angles that are less than 180° between the directions of these moments and the direction of the torque. The existence of the angles results in these magnetic moments are easier to be flipped than those in the body of S2.

**Figure 4:**
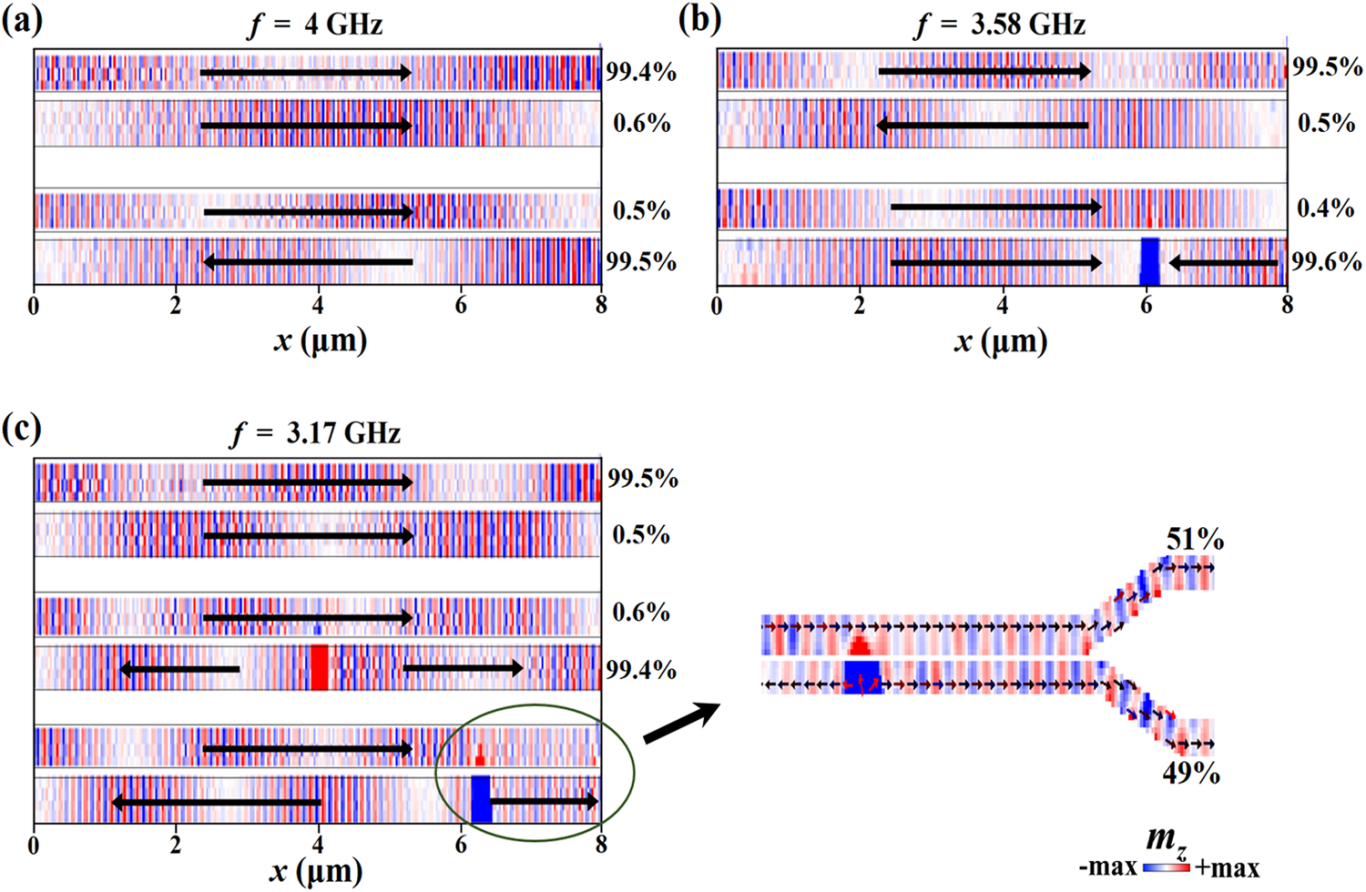
Switching of the functionality and operating frequency with magnetic configuration. The SW amplitude is shown by color maps. The arrows show the direction of static magnetization. The percentages indicate the ratio of the output powers in two waveguides. Inset: functionality of power divider resulted by coupling length *L* sudden change.

Finally, we demonstrate that the functionality and operating frequency of the coupler can be dynamically switched using method proposed above. Figure 4(a) shows the color maps of the SW amplitude in the proposed device with parallel and antiparallel magnetic configurations. The frequency of the excited SW is 4 GHz. In the initial state, the magnetic configuration is parallel. After propagating a distance of 2 *L*, the entire SW power is transferred back to S1. The coupler acts as a simple transmission line. Then shutting off the SW excitation, electrical current is applied on all four metal strips in the +y direction with a time duration of 86 ns, after 70 ns natural relaxation, the configuration is inverted to antiparallel. The *L* for the SW meets the condition *L*_*w*_ = 3*L* in this case. Therefore, the power is totally transferred to S2 at the end. The coupler acts as a connector of units in magnonic circuits.

For a specific operating frequency, the results presented above show that the functionality of the coupler can be switched by inverting whole magnetic configuration using SOT. However, an inversion of selected part of magnetic configuration opens an additional degree of freedom for the operating frequency of the proposed device. A simple instance of such an application is depicted in Fig. 4(b,c) (Table 1 shows explicit functionalities). As shown in Fig. 4(b), First, the current is applied on all four strips in the +y direction, the configuration is inverted to completely antiparallel. For a SW at 3.58 GHz, the *L* satisfies *L*_*w*_ = 4*L*, so the coupler acts as a transmission line. But for the *L* in totally parallel state, *L*_*w*_ < 3*L*. Thence, the 3.58 GHz is not an operating frequency of parallel-state coupler. To solve this issue, current is applied on all the strips except for number 4 in the −y direction. The configuration is inverted to a hybrid state of 3/4 parallel and 1/4 antiparallel. It is equivalent to a 6-μm-long parallel-state coupler connected to a 2-μm-long antiparallel-state coupler. Since the *L* in antiparallel configuration is much shorter, the entire SW power can be transferred to S2. Thus, the 3.58 GHz becomes an operating frequency of the coupler after this modification. Similar results are shown in Fig. 4(c). For the SW at 3.17 GHz, the *L* satisfies *L*_*w*_ = 4*L* in parallel configuration. Then current is applied on the strips numbered 1 and 2 in the +y direction. It results in a hybrid state of semi-antiparallel and semi-parallel, and the SW power can be completely transferred to S2. Finally, we apply current on the strip numbered 3 in the +y direction only. After that, a state of 3/4 antiparallel and 1/4 parallel is formed. The SW power can be equally divided and output via the ports in this case, i.e. the coupler acts as a power divider. The 3.17 GHz becomes another new operating frequency.

**Table 1:** The truth table of the proposed directional coupler.

f (GHz)	Configuration	Output_S1_	Output_S2_
4	Parallel	1	0
	Antiparallel	0	1
3.58	Antiparallel	1	0
	1/4 Antiparallel	0	1
3.17	Parallel	1	0
	1/2 Antiparallel	0	1
	3/4 Antiparallel	1	1

It is worthy to note that the functionality of power divider here is not realized by adjusting coupling length *L* to meet condition *L*_*w*_ = (*n* + 1/2)3*L*^14^, but the effect of sudden change in *L*. The inset of Fig. 4(c) shows the principle. Before the SW power propagating in S1 pass by the position where *L* suddenly change, about 50% of the power has been transferred to S2. When the SWs in S1 and in S2 pass by the position, it is equivalent to input the same SW signals simultaneously on both ports of a coupler in parallel state. Consequently, the periodic transfer of the SW power is destroyed and the powers in both waveguides will no longer be transferred. This phenomenon will not happen when the SW passes by the *L*-sudden-change position, the power in S1 has not begun to be transferred or has been completely transferred to S2 (see Fig. 4(b) and the middle figure of 4(c)). In general, when designing a proposed coupler, this phenomenon should be avoided, but it can also be utilized for the realization of arbitrary SW power divider. In practice, long hours of SW transportation will lead to the motion of domain wall. Thereby, it will result in the proportional change of different configuration regions that would destroy the functionality of the coupler (see the bottom figure of 4(c), but in this case, the functionality has not changed). This problem can be addressed by the introduction of patterning notches along the edges of the waveguide at the positions of the gaps between metal strips^31–33^. After modifying the geometry, the domain wall can be pinned and this do not lead to the destruction of functionality (see Supplementary Fig. S1).

In summary, we propose a reconfigurable nanoscale SW directional coupler based on current-induced SOT. Using micromagnetic simulations, we demonstrate that the functionality and operating frequency of the coupler can be dynamically switched in 156 ns by inverting the whole or part of magnetic configuration of one of the coupled waveguides utilizing SOT. The optimal value of switching current density is determined to be 12 × 10^11^ A/m^2^. Using effect of sudden change in coupling length, the functionality of SW power divider can be realized. Our proposal opens the path to efficient, bias-free, current-controlled reconfigurable connection that can be used in future magnonic integrated circuits.

## Methods

The dipolar-coupled waveguides studied are sketched in Fig. 1. Two 30-nm-thick bent YIG waveguides with width of 100 nm and the working length of 8 μm are studied in our works. Four identical β-Ta strips which are separated by a gap with width of *g* = 20 nm from each other are placed on the S2 waveguide. The material parameters of β-Ta and YIG nanometer-thick film used in simulations are from refs^26,33,34^. For 8 nm-thick β-Ta, the spin-Hall angle is 0.12^26^. For YIG, saturation magnetization *M*_s_ = 1.4 × 10^5^ A/m, exchange constant *A*_ex_ = 3.5 × 10^−12^ J/m, Gilbert damping α = 2 × 10^−4^ ^34,35^. The cell size is 10 × 10 × 10 *nm*^3^. According to previous study, the existence of β-Ta layer does not obviously increase the damping in an adjacent magnetic film^26^. Our additional simulations also have shown that the functionality of coupler does not change when the damping in S2 is increased to 6 × 10^−4^ (see Supplementary Fig. S2). Thence, in our simulations the damping in S2 is as same as that in S1, i.e. 2 × 10^−4^. It should be noted that although the using of β-Ta can solve the problem of the damping increase in YIG waveguides, the high resistivity of β-Ta (about 190 μΩ•cm)^26^ may lead to an energy efficiency problem. The Joule heat from β-Ta layer will heat the YIG layer. Since the overheat will lead to a change in magnetic parameters of YIG film, this issue limits the frequency of the magnetic configuration switching and consequently, the frequency of the functionality switching. To address this issue, a new material with high spin Hall angle, low resistivity and the property that does not cause an increase of the damping in the adjacent magnetic film should be developed. The micromagnetic simulations are performed using micromagnetic package MuMax3^36^, which is capable of solving the Landau-Lifshitz-Gilbert equation with Slonczewski-like STT term. Although the origins of STT and SOT are different, their effects are similar. Therefore, in micromagnetic simulations, SOT can be simulated by STT term after a numerical conversion^37,38^. The STT term ${\tau }_{STT}(r) \sim ({\theta }_{SH}g{\mu }_{B}J)/(2e{t}_{YIG}{M}_{s})\,$^37^, where θ_SH_ is spin-Hall angle, *g* is Lander factor, *μ*_*B*_ is Bohr magneton, *J* is charge current density, *e* is electron charge, *t*_*YIG*_ and *M*_*s*_ is the thickness and saturation magnetization of YIG film, respectively. To excite a propagating SW in S1, a sinusoidal magnetic field *b*_*y*_ = *b*_0_ sin(2*πft*) with the amplitude *b*_0_ = 1 mT produced by 20 nm width microstripe antenna is applied at the edge of S1.

## Supplementary information

Supplementary Material
