# Supplementary material for: Reconfigurable nanoscale spin-wave directional coupler using spin-orbit torque
Source: Sci Rep. 2019 May 8;9:7093. doi: 10.1038/s41598-019-43597-6 (PMC6506528; doi:10.1038/s41598-019-43597-6)
Supplement: Supplementary file 1 — Supplementary Material [file 41598_2019_43597_MOESM1_ESM.pdf]

Supplementary Material: Reconfigurable nanoscale spin-wave directional coupler using spin-orbit torque

Zhiwei Ren, Shuang Liu, Lichuan Jin, Tianlong Wen, Yulong Liao, Xiaoli Tang, Huaiwu Zhang & Zhiyong Zhong*

State Key Laboratory of Electronic Thin Films and Integrated Devices, University of Electronic Science and Technology of China, Chengdu, 611731, China

In practice, long hours of spin wave (SW) transportation will lead to the motion of domain wall which is formed between different magnetic configuration regions in the waveguide. Thereby, it will result in the proportional change of different configuration regions that would destroy the functionality of the coupler. This problem can be solved by the introduction of patterning notches along the edges of the waveguide at the positions of the gaps between metal strips[1-3]. Without loss of generality, we performed the simulation in the case of 1/2 antiparallel and 1/2 parallel magnetic configuration only. The frequency of excited SW is 3.17 GHz. In order to increase the accuracy, the cell size was reduced to $5\times5\times10$ nm^3. As shown in Fig S1(a), two identical triangular notches are made at the position x = 4 μm with the depth of 15 nm and the width of 30 nm. By comparing with Fig S1(b) and (c), one can draw a conclusion that the existence of the notches do not lead to a substantial impact on the functionality of the coupler. The domain wall is pinned and does not move in 500 ns. Thus, the introduction of notches can ensure long-term normal operation of the coupler.

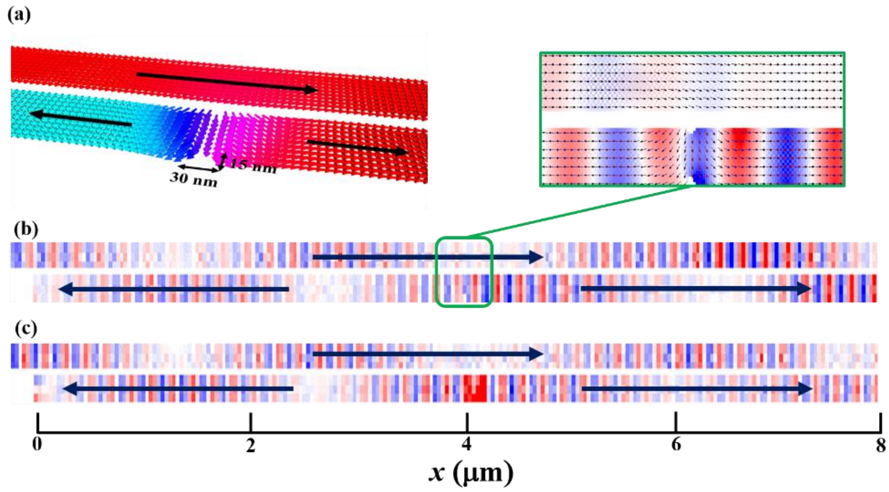

Figure S1. (a) Part of the magnetic configuration of the coupled waveguides with two identical triangular notches at the position x = 4 μm. The depth and width of the notches is 15 nm and 30 nm, respectively. (b) The color map of the SW amplitude when there are notches on the edges of the waveguide. The inset shows the detail. The frequency of the SW is 3.17 GHz. (c) The color map of the SW amplitude when there is not any notch on the edge of the waveguide. The frequency of the SW is 3.17 GHz.

In our works, according to previous study on CoFeB/β-Ta system[4], the micromagnetic simulations were performed under the hypothesis that the existence of β-Ta strips will not increase the damping in YIG ($Y_3Fe_5O_{12}$) waveguides. However, due to the spin pumping and other interfacial effects[5], the damping of YIG may increase slightly in practice. Therefore, we performed an additional simulation, in which the Gilbert damping of the waveguide covered by β-Ta strips was set to three times of that of the other waveguide, i.e. $\alpha_{S2} = 6\times10^{-4}$, and there are two identical triangular notches on the edges the waveguide. As shown in Fig S2, there is no difference in functionality between the two cases.

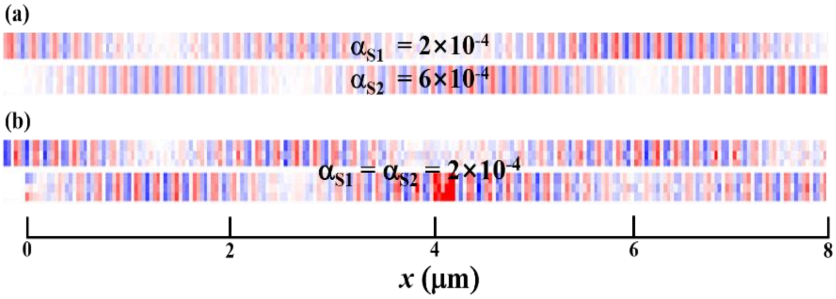

Figure S2. (a) The color map of SW amplitude when the damping is set to 2×10^{-4} and 6×10^{-4} for the two waveguides, respectively. There are two identical notches on the edge of the waveguide covered by metal strips at the position $x = 4$ μm. The frequency of the SW is 3.17 GHz. (b) The color map of SW amplitude when the damping of both of the waveguides is 2×10^{-4}. There is not any notch on the edge of the waveguide. The frequency of the SW is 3.17 GHz.

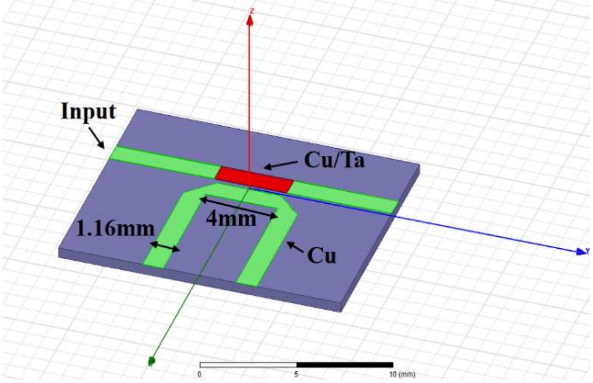

Figure S3. The schematic model of the microwave directional coupler used in the comparative test. The width of the microstrip lines is 1.16 mm. The working length is 4 mm. the thickness of Cu microstrip lines and Ta layer is 0.01778 mm and 0.001 mm, respectively.

Finally, although micromagnetic simulations were used in previous studies on similar hybrid systems and showed agreement with experiments (Refs. 6, 7, 8), there is still the possibility that the existence of the additional Ta layer leads to a disturbance on the operation of the coupler due to the RF propagation modulation. Therefore, we performed an electromagnetic simulation using ANSYS HFSS[9] (High Frequency Simulator Structure). Since the simulation of pure ferrite waveguides is difficult in this

framework, as a substitute, we performed a comparative test between traditional microwave directional coupler consists of Cu microstrip lines and Cu/Ta bilayer microstrip lines.

Based on the observation of the curves of S(3,1) parameter (Figs S4 (a) and (b)), the results show that the RF propagation modulation do decrease the coupling degree of the coupler slightly, but do not destroy the functionality. Thence, we believe that the introduction of Ta layer on the top of one of the coupled waveguides will not destroy the operations of our proposal.

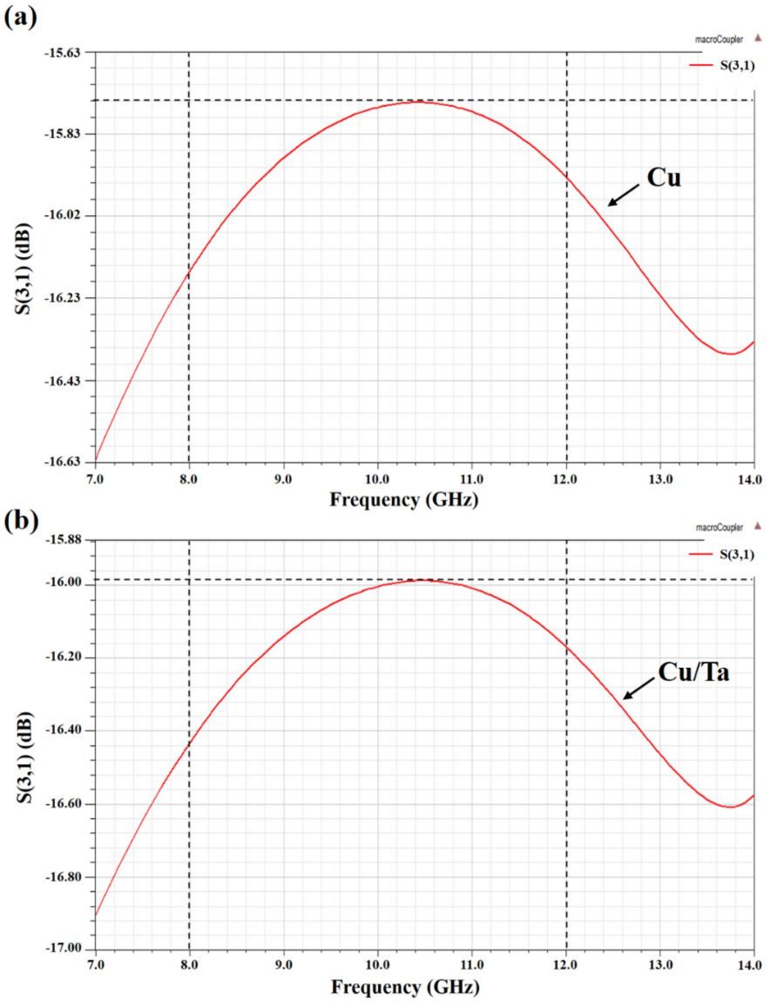

Figure S4. (a) The curve of S(3,1) parameter in the case of pure Cu micostrip lines. (b) The curve of S(3,1) parameter in the case of Cu/Ta bilayer microstrip lines.

1. Dorothée, P., Jausovec, A. V., Read, D. & Cowburn, R. P. Domain wall pinning and potential landscapes created by constrictions and protrusions in ferromagnetic nanowires. *J. Appl. Phys.* **103**, 9310 (2008).
2. Parkin, S. S. P., Hayashi, M. & Thomas, L. Magnetic domain-wall racetrack memory. *Science.* **320**, 190-194 (2008).
3. Atkinson, D., Eastwood, D. S. & Bogart, L. K. Controlling domain wall pinning in planar nanowires by selecting domain wall type and its application in a memory concept. *Appl. Phys. Lett.* **92**, 585

(2008).
4. Liu, L. *et al*. Spin-torque switching with the giant spin hall effect of tantalum. *Science.* **336**, 555-558 (2012).
5. Tserkovnyak, Y., Brataas, A. & Bauer, G. E. W. Enhanced gilbert damping in thin ferromagnetic films. *Phys. Rev. Lett.* **88**, 117601(2002).
6. Pirro, P. *et al*. Spin-wave excitation and propagation in microstructured waveguides of yttrium iron garnet/pt bilayers. *Appl. Phys. Lett.* **104**, 012402 (2014).
7. Ulrichs, H., Demidov, V. E. & Demokritov, S. O. Micromagnetic study of auto-oscillation modes in spin-hall nano-oscillators. *Appl. Phys. Lett.* **104**, 467-266 (2014).
8. Bhowmik, D. *et al*. Deterministic domain wall motion orthogonal to current flow due to spin orbit torque. *Sci. Rep.* **5**, 11823 (2015).
9. "High Frequency Structure Simulator (HFSS) Ver. 15," Ansoft, (2014). Available: http://www.ansys.com.
